# Therapeutic effect of autologous bone grafting with adjuvant bone morphogenetic protein on long bone nonunion: a systematic review and meta-analysis

**DOI:** 10.1186/s13018-022-03185-3

**Published:** 2022-06-03

**Authors:** Chengxin Xie, Chenglong Wang, Yu Huang, Qinglong Li, Xin Tian, Wenwen Huang, Dong Yin

**Affiliations:** 1 Department of Orthopedics, Taizhou Hospital of Zhejiang Province & Taizhou Hospital Affiliated to Wenzhou Medical University, No.150 Ximen Road, Linhai, 317000 Zhejiang Province China; 2 Department of Joint Surgery and Sports Medicine, Guangxi Academy of Medical Sciences & The People’s Hospital of Guangxi Zhuang Autonomous Region, No.6 Taoyuan Road, Nanning, 530001 Guangxi Zhuang Autonomous Region China; 3 Department of Traumatic Surgery & Microsurgery & Hand Surgery, Guangxi Academy of Medical Sciences & The People’s Hospital of Guangxi Zhuang Autonomous Region, No.6 Taoyuan Road, Nanning, 530001 Guangxi Zhuang Autonomous Region China

**Keywords:** Bone morphogenetic protein, Autologous bone graft, Nonunion, Long bone, Meta-analysis

## Abstract

**Background:**

The recombinant human bone morphogenetic protein (rhBMP) is a common graft substitute for treating cases of long bone nonunion. However, the feasibility of combining an autologous bone graft (ABG) with rhBMPs remains uncertain. Thus, this systematic review and meta-analysis aimed to evaluate the synergistic effect of ABG and rhBMPs on the healing of long bone nonunion.

**Methods:**

A systematic literature search was performed on PubMed, Web of Science, Cochrane Library, and China National Knowledge Infrastructure. Two authors independently screened the studies, extracted data, and assessed the quality of the trials. Statistical analyses were performed using Stata 12.0.

**Results:**

Of the 202 citations, five studies involving a total of 394 cases met the eligibility criteria; thus, they were included in this study. The pooled data revealed no significant differences among the groups in terms of postoperative healing rate (risk ratio [RR] = 1.01, 95% confidence interval [CI] = 0.96–1.06, *P* = 0.744), healing time (standardised mean difference =  − 0.20, 95% CI = − 0.95–0.56, *P* = 0.610), and pain (RR = 1.44, 95% CI = 0.25–8.29, *P* = 0.681). The combination of ABG and rhBMPs resulted in good limb function (RR = 1.31, 95% CI = 1.04–1.66, *P* = 0.023).

**Conclusions:**

The combination of ABG and rhBMPs did not result in the healing of long bone nonunion and pain reduction. Nevertheless, it conferred good limb function. Thus, the findings in this study are insufficient to support the use of rhBMPs as an adjuvant to ABG.

## Background

A nonunion fracture results in significant morbidity, prolonged hospital stay, and increased expenses [1]. Fractures other than the nonunion ones typically heal within 20 weeks. However, a nonunion fracture presents with incomplete healing even six months after the injury [2]. The incidence of delayed union or nonunion of long bone fractures is estimated to be between 2.5% and 46%, depending on the location and severity of damage to the bone, soft tissue, and vascular structures [3]. Treatment of nonunion fractures involves mechanical fixation and biological repair, which often requires an autologous bone graft (ABG), to achieve osteogenic augmentation [4]. Although several bone graft substitutes are available for a nonunion repair, ABG remains the gold standard for treatment because of its accessibility and the abundance of progenitor cells and growth factors in patients [3–5].

Recombinant human bone morphogenetic proteins (rhBMPs) are a family of soluble bone matrix glycoproteins that induce the migration, proliferation, and differentiation of undifferentiated mesenchymal stem cells to form osteoblasts and chondroblasts [1]. With the development of synthetic implants and graft adjuvants, the combination of ABG and rhBMPs for enhancing fracture healing has gained popularity. Healing rates as high as 92% have been reported in persistent nonunion patients after undergoing rhBMP treatment with or without ABG [6]. A previous meta-analysis on this topic reported that rhBMPs and ABG yielded similar healing rates for tibial nonunion fracture cases [7]. However, there has been no meta-analysis on the use of ABG with or without adjuvant rhBMP for the treatment of long bone nonunion. Thus, this study aimed to determine whether rhBMPs worked synergistically with ABG to enhance bone healing in cases of long bone nonunion.

## Methods

This study adheres to the Preferred Reporting Items for Systematic Reviews and Meta-Analyses (PRISMA) guidelines [8].

### Search strategy

We systematically searched major databases, which included PubMed, Web of Science, Cochrane Library, and China National Knowledge Infrastructure (CNKI), without any language restriction for articles published between January 2001 and May 2021. The search strategy involved free text terms relevant to bone morphogenetic protein, autologous bone grafting, and nonunion. The terms and Boolean operators used were as follows: (bone morphogenetic protein OR BMP OR osteogenic protein-1 OR OP-1 OR Osigraft) AND (autologous bone grafting OR ABG) AND (nonunion OR delayed union). The search strategies were adjusted for each database. Furthermore, three authors manually screened the references of the included articles to identify additional eligible trials.

### Eligibility criteria

The inclusion criteria were as follows: (1) the participants of the study were patients aged 18 years or older with long bone nonunion; (2) the patients in the treatment group received therapy with combined ABG and rhBMPs; (3) the patients in the control group were treated with ABG; (4) the postoperative healing rate and healing time were the primary outcomes of the study; and (5) the study was a randomised controlled trial or cohort study. In contrast, the exclusion criteria were as follows: (1) study with clearly erroneous or incomplete data or data duplicated from another study; (2) literature without full text; and (3) animal studies. Studies that satisfied the inclusion and exclusion criteria were included in this systematic review and meta-analysis.

### Data extraction and quality assessment

Two authors independently retrieved and screened the articles according to the inclusion and exclusion criteria, extracted the data, and assessed their quality. We collected data on the study characteristics (first author’s name, year of publication, country of origin, study design, sample size, and follow-up duration), participant demographics (sex, age, and nonunion time), interventions (type of rhBMPs administered), outcome measures (healing rate, healing time, excellent and good rate of function, and pain), and other information needed to assess the risk of bias and methodological quality. The Newcastle–Ottawa Scale (NOS) was used to evaluate the quality of cohort studies [9]. The score of the items that are indicative of study quality varied from 0 to 9 according to the following categories: selection of cohorts (four items), comparability of cohorts (one item), and assessments of outcomes (three items). A maximum of one star was awarded for each item in the selection of cohorts and assessments of outcome categories, while two stars were awarded for an item in comparability of cohorts. A study was considered as being of high quality if it received a score 7 or more (☆ ≥ 7), moderate when it received a score of 4–6 (4 ≤ ☆ ≤ 6), and poor when it received a score of 0–3 (☆ ≤ 3). Any disagreement between assessors was resolved by a group discussion with a third author.

### Statistical analyses

All statistical analyses were performed using Stata 12.0. The statistical heterogeneity across studies was quantified by calculating the *I*^2^ statistic. An *I*^2^ value of > 50% was considered to be significantly inconsistent. The random-effects model was used to compare heterogeneous studies, while a fixed-effects model was used to compare homogenous studies. The risk ratio (RR) was calculated for dichotomous variables, while the standardised mean difference (SMD) was calculated for continuous variables in each study. The 95% confidence interval (CI) was determined to calculate the effect size. In addition, sensitivity analysis was conducted using the trim-and-fill method. The presence of publication biases was assessed using the Begg’s funnel plots and Egger’s test. All *p values* were two-sided, and statistical significance was set at *P* < 0.05.

## Results

### Literature search results

**Fig. 1:**
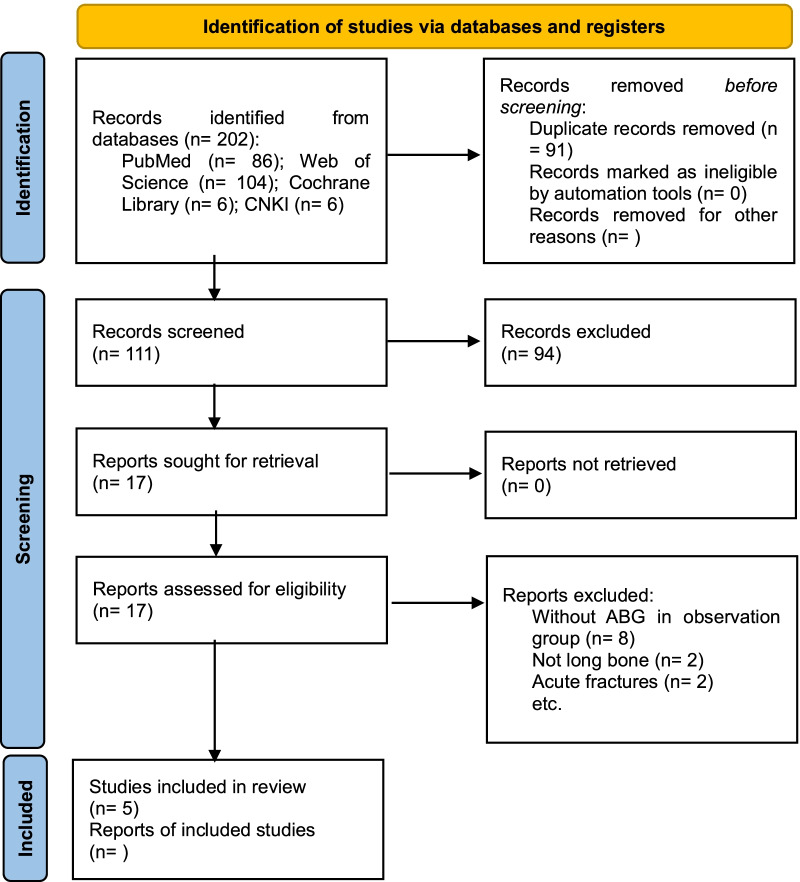
A flow diagram of the literature search

As shown in Fig. 1, 202 potentially relevant articles were identified through a systematic search. After duplicates were removed, we screened the titles and abstracts of 111 articles and excluded the irrelevant ones. We then reviewed the full text of the remaining 17 articles and excluded studies based on our criteria. Finally, five studies [10–14] that fulfilled the predefined selection criteria for this meta-analysis were included.

### Description of included studies

The baseline characteristics of the five studies are summarised in Table 1 [10–14]. The five articles were cohort studies conducted in three countries and published between 2014 and 2019. Most of these had studied small sample sizes. The combined total of the nonunion cases in the five articles were 394. These cases involved the tibia, femur, forearm, humerus, and clavicle. Notably, one of these studies [10] involved a very small number (4.4% in the observation group and 4.0% in the control group) foot–ankle nonunion cases; however, we retained this study to expand the sample size of our meta-analysis.

**Table 1:** Characteristics of the included studies

Author	Year	Country	Study design	Intervention	Sample size (*n*)	Age (year)	Women [*n* (%)]	Nonunion		Follow-up time (months)	Outcome
					Observation/control			Time (months)	Site		
Takemoto [10]	2014	USA	Cohort study	ABG + rhBMP-2	68 50	47.4 43.4	28 (41%) 15 (30%)	NR	(A)(B)(C) (D)(E)(F)	12	①②
Von Ruden(a) [11]	2016	Germany	Cohort study	ABG + (rhBMP-2 or rhBMP-7)	24 25	43 45	2 (8%) 9 (36%)	11	(C)	6–54	①③④
VonRuden(b) [12]	2016	Germany	Cohort study	ABG + (rhBMP-2 or rhBMP-7)	28 45	50 49	13 (46%) 14 (31%)	11 13	(E)	6–52	①②
Hackl [13]	2017	Germany	Cohort study	ABG + rhBMP-7	62 50		24 (39%) 13 (26%)		(A)(B)(D)	> 12	①②
(a)Humerus					13 9	49 51	6 (46%) 3 (33%)	14.8			
(b)Femur					28 13	55.2 42.4	11 (39%) 2 (15%)	11.7			
(c)Tibia					21 28	51.6 42.3	7 (33%) 8 (29%)	10.3			
Liu [14]	2019	China	Cohort study	ABG + rhBMP-2	22 20	42.25 41.57	9 (41%) 7 (35%)	9.37 9.24	(A)(B)(C) (D)	6–24	①②③④

*ABG* autologous bone graft, *rhBMP* recombinant human bone morphogenetic protein, *(A)* tibia, *(B)* femur, *(C)* forearm, *(D)* humerus, *(E)* clavicle, *(F)* others, *①* healing rate, *②* healing time, *③* excellent and good rate of function, *④* pain

As shown in Table 2, the cohort studies had a high methodological quality according to the NOS. This conclusion was based on the total scores of these studies, which varied from six to nine.

**Table 2:** Methodological quality of the cohort studies according to the NOS

Study	Selection of cohorts	Comparability of cohorts	Assessment of outcome	Total score
Takemoto [10]	☆☆☆	☆	☆☆☆	7
Von Ruden(a) [11]	☆☆☆☆	☆☆	☆☆☆	9
VonRuden(b) [12]	☆☆☆☆	☆☆	☆☆☆	9
Hackl [13]	☆☆☆☆	☆☆	☆☆☆	9
Liu [14]	☆☆☆	☆	☆☆	6

### Outcomes

#### Postoperative healing rate

**Fig. 2:**
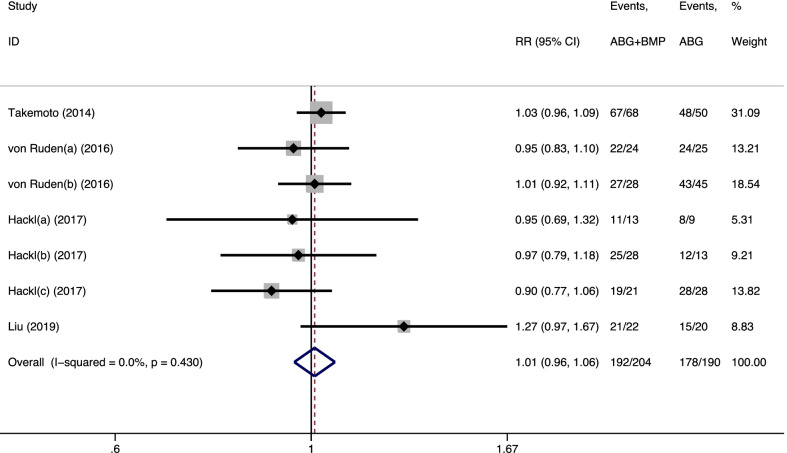
Forest plot of the postoperative healing rate

All five studies (seven trials) [10–14] reported data on the postoperative healing rate. There were 204 and 190 cases in the observation and control groups, respectively. No significant statistical heterogeneity was observed among the trials (*I*^2^ = 0.0%, *p* = 0.430). The pooled results derived from the fixed-effects model are presented in Fig. 2. There was no statistically significant difference between the two groups (RR = 1.01, 95% CI = 0.96–1.06, *p* = 0.744).

#### Postoperative healing time

**Fig. 3:**
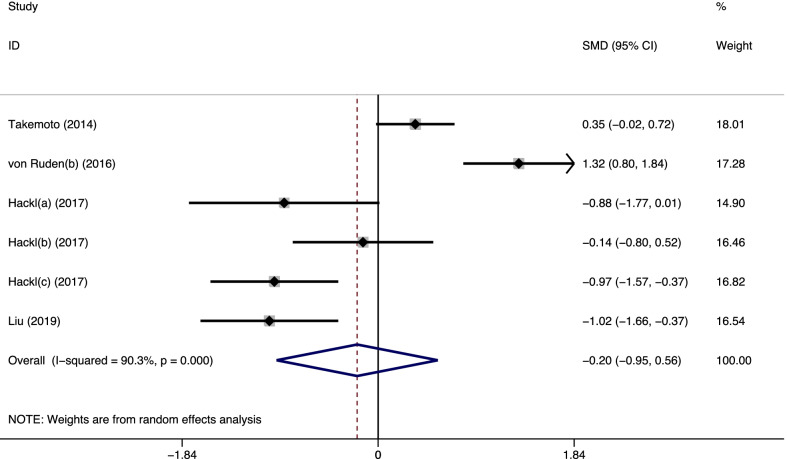
Forest plot of the postoperative healing time

Four of the five studies (six trials) [10, 12–14] provided data on postoperative healing time. As shown in Fig. 3, there was a significant statistical heterogeneity among the trials (*I*^*2*^ = 90.3%, *p* < 0.001); thus, a random-effects model was used to pool the results. No significant difference was observed between the groups (SMD = − 0.20, 95% CI = − 0.95–0.56, *p* = 0.610). Analysis of the causes of heterogeneity revealed that all cases in Von Ruden’s study (b) [12] were of clavicle nonunion. The differences of biomechanical and biological properties between the clavicle and limb were significant, which might have introduced additional heterogeneity to the pooled sample.

#### Excellent and good rate of limb function

**Fig. 4:**
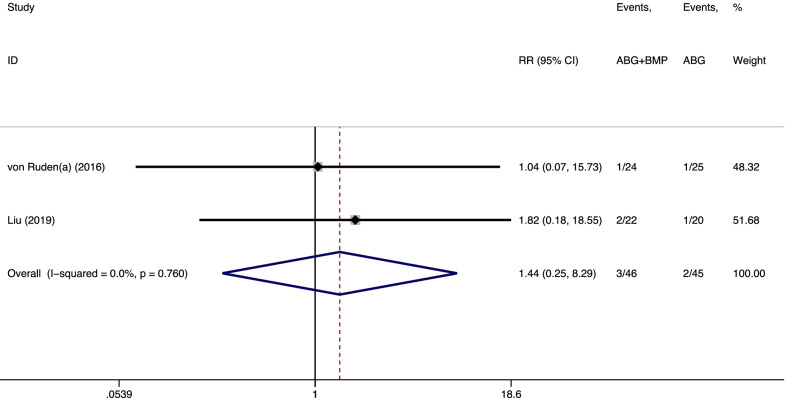
Forest plot of those with excellent and good rates of function

Excellent limb function was achieved in only two studies [11, 14]. As shown in Fig. 4, a fixed-effects model was applied because no statistical heterogeneity was found between the trials (*I*^*2*^ = 0.0%, *p* = 0.777). The pooled results showed statistically significant differences between the groups (RR = 1.31, 95% CI = 1.04–1.66, *p* = 0.023), indicating that the addition of rhBMPs might have improved postoperative limb function.

#### Postoperative pain rate

**Fig. 5:**
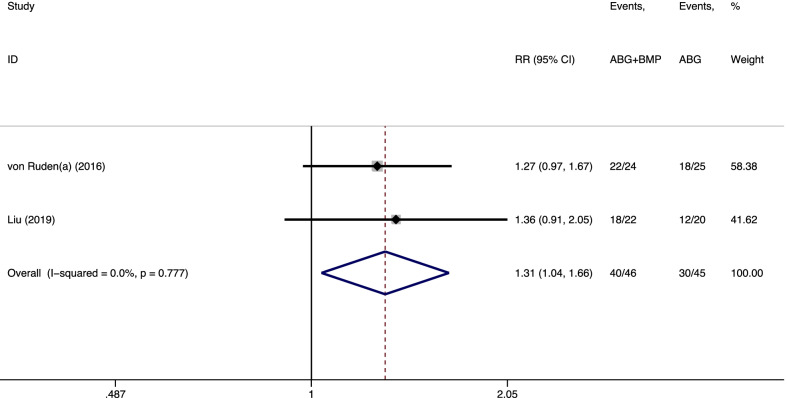
Forest plot of the postoperative pain rate

Only two studies [11, 14] reported the data on postoperative pain rate. As shown in Fig. 5, the pooled results revealed no significant difference (RR = 1.44, 95% CI = 0.25–8.29, *p* = 0.681) between the observation and control groups. There was no heterogeneity between the two studies (*I*^*2*^ = 0.0%, *p* = 0.760).

### Sensitivity analysis

**Fig. 6:**
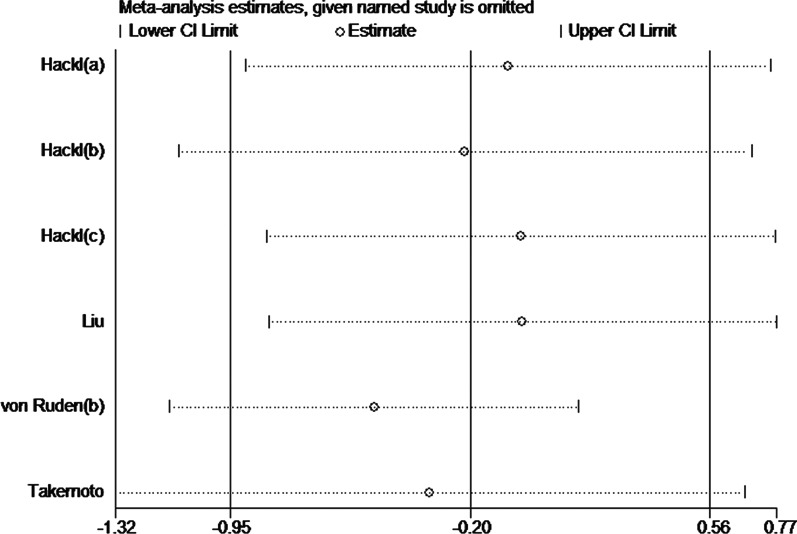
Sensitivity analysis of the postoperative healing time via the trim-and-fill method

Given that a significant heterogeneity existed in the postoperative healing time (*I*^2^ = 90.3%, *p* < 0.001), the trim-and-fill method was used for the sensitivity analysis. The results are shown in Fig. 6. Furthermore, we manually removed one trial from the pooled analysis each time and did not find significant changes in SMDs or 95% CIs. Thus, our conclusions are relatively robust and credible.

### Publication bias

**Fig. 7:**
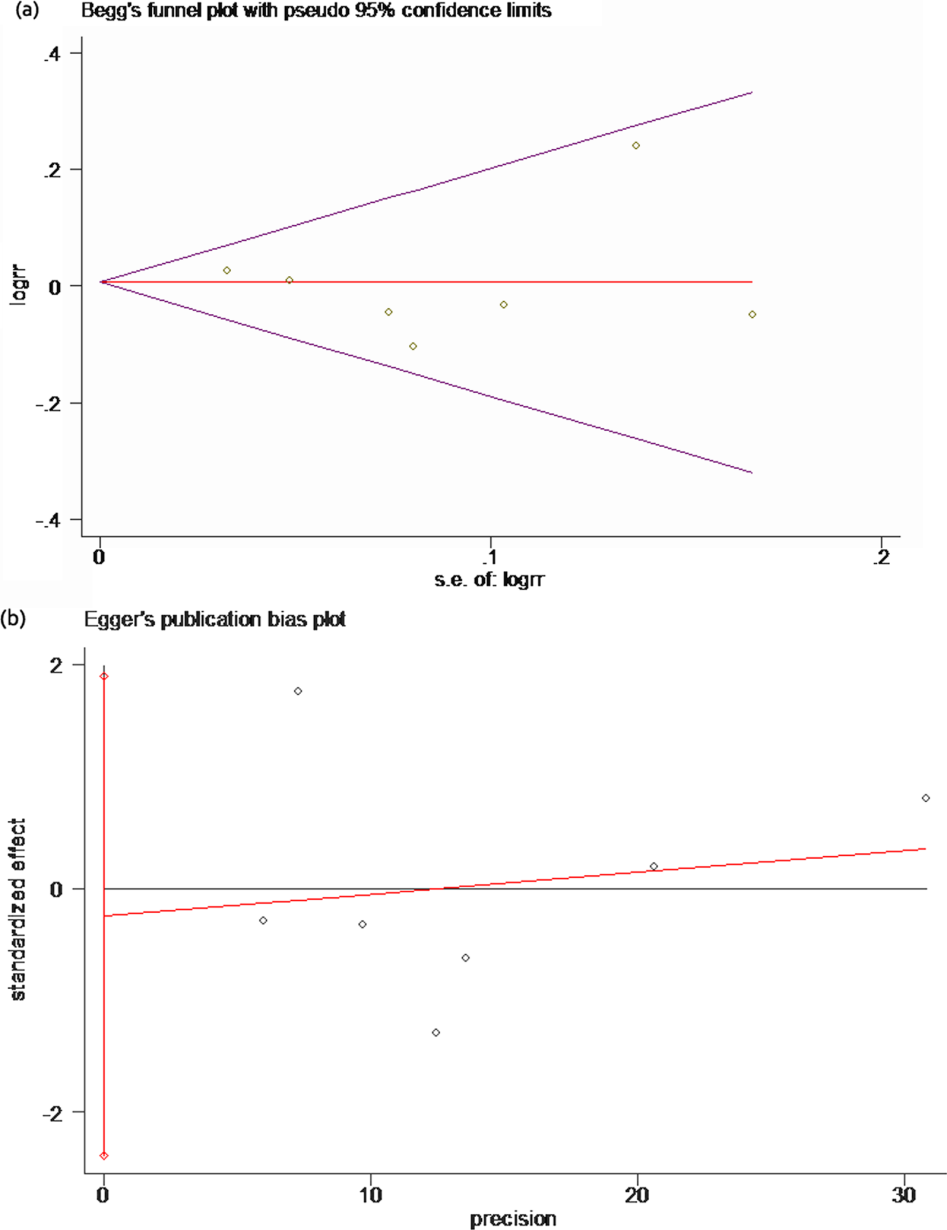
Publication bias of the postoperative healing rate. **a** Begg’s funnel plot; **b** Egger’s bias plot

The sample size of postoperative healing rate was relatively large; therefore, we performed the Begg’s test and Egger’s test to determine whether there is publication bias. As shown in Fig. 7, there was no significant publication bias across studies, as determined by both tests (Begg’s test, *p* = 0.881, Fig. 7a; Egger’s test, *p* = 0.778, Fig. 7b).

## Discussion

The incidence of nonunion is as follows: radius, 5%; ulna, 7%; clavicle, 7.5%; humerus, 9%; femur, 12.5%; tibia, up to 45% [11, 15, 16]. Surgery in these cases of long bone nonunion is often challenging because of several associated risks [16, 17]. Current strategies for promoting bone healing focus on the topical application of growth factors, such as rhBMPs, which are considered the most potent osteo-inductive agents [18]. Extensive clinical data demonstrated the increased potential of rhBMPs to induce healing of long bone nonunion relative to standard treatments, such as ABG. Furthermore, most studies reported equivalent or superior results with rhBMPs alone (with their carrier matrix) than with standard treatment, in terms of healing acceleration, clinical outcome, and radiographic consolidation [19]. In contrast, several studies reported that the unique advantage of bone induction was not superior to that of ABG [20–22].

This is the first meta-analysis to investigate whether ABG combined with rhBMPs yielded a synergistic effect on long bone nonunion. However, this treatment did not yield an increased healing rate and shortened healing time of long bone nonunion. As the gold standard for bone graft material, autologous bones stimulate osteo-genesis, osteo-induction, and osteo-conduction in a nonunion site. These effects are the three important biological factors of bone healing according to the "diamond concept" proposed by Giannoudis et al. [23]. Under these conditions, additional application of rhBMPs seems unnecessary. Furthermore, our results show that the use of rhBMPs could not reduce the incidence of postoperative pain despite the advantages of rhBMPs as an alternative to ABG if patients preferred avoiding pain in donor sites. Although nonunion fractures vary greatly among their different anatomical locations and degrees of injury, the ultimate goal of operative treatment is to restore the bone as completely as possible. Fortunately, we observed positive results in favour of the addition of rhBMPs. This treatment regimen resulted in better limb function. However, this outcome variable was pooled from only two studies with 91 cases, and thus still lacks reliability.

Successful healing of long bone nonunion depends more on a standard protocol involving fibrous debridement, restoration of axis, length and torsion, stable fracture fixation, repair of soft tissue, and augmentation with an ABG rather than on the adjunctive use of rhBMPs [13, 20]. However, potential causes of nonunion should be addressed, such as infection, serious bone defects, excess motion, inadequate vascularity, and other systemic factors [17]. In addition, the cost and cost-effectiveness of rhBMPs should be considered because they are used as off-label indications. In Italy, economic studies supported the early use of rhBMPs as a more cost-effective strategy than ABG for treating severe cases of bone and soft tissue damage. These studies considered the costs of prolonged hospitalisation, medication, repeated surgical failure, and disability [24, 25]. Moreover, the combination of rhBMPs and allografts was shown to be a valid alternative to ABG, leading to equivalent beneficial effects and the avoidance of the drawbacks related to invasive autologous bone harvesting procedures[19, 20]. However, since the synergistic effect of rhBMPs and ABG is not desired, the medical costs are higher if rhBMPs are used adjunctively. Finally, a multitude of factors ultimately contributes to the outcome and the different conditions requiring specific treatments. All of these may obscure the benefits of rhBMPs.

The results of this meta-analysis should be interpreted in light of its limitations. First, the size of each individual study was small. Furthermore, all of the included studies were non-randomised controlled trials that would decrease the level of evidence. Second, the combined analysis of several different long bones with unequal biomechanical and biological properties might have weakened the applicability of the conclusions. Third, the type, carrier, and dose of rhBMPs varied among the studies; thus, these factors were reported to have discrepancies in the results [26–28]. Fourth, many independent variables could have affected the nonunion fractures. Subtle differences inherent to the treatment of each type of nonunion are inevitable. It was difficult to draw an affirmative conclusion regarding the real potential of rhBMP therapy for the treatment of long bone nonunion. In the future, researchers should pool more studies with larger samples and conduct subgroup analyses for individual long bones, specified nonunion type, and rhBMP type.

## Conclusions

This meta-analysis showed that the synergistic effect of an ABG and rhBMPs was not desirable for the healing of long bone nonunion and pain reduction. Although better limb function was observed, current evidence remains insufficient to support the use of rhBMPs as an adjuvant to ABG. More well-designed studies are needed to confirm the reliability of our results.

## Data Availability

The generated and analysed datasets are available from the corresponding author upon reasonable request.

## Abbreviations

rhBMP: Recombinant human bone morphogenetic protein
ABG: Autologous bone graft
CNKI: China National Knowledge Infrastructure
RR: Risk ratio
SMD: Standardised mean difference
CI: Confidence interval
NOS: Newcastle–Ottawa Scale

## References

[1] Marupanthorn K, Tantrawatpan C, Kheolamai P, Tantikanlayaporn D, Manochantr S (2017). Bone morphogenetic protein-2 enhances the osteogenic differentiation capacity of mesenchymal stromal cells derived from human bone marrow and umbilical cord. Int J Mol Med.

[2] Garrison KR, Shemilt I, Donell S, Ryder JJ, Mugford M, Harvey I, Song F, Alt V (2010). Bone morphogenetic protein (BMP) for fracture healing in adults. Cochrane Database Syst Rev.

[3] Sen M, Miclau T (2007). Autologous iliac crest bone graft: should it still be the gold standard for treating nonunions?. Injury.

[4] Lin K, VandenBerg J, Putnam SM, Parks CD, Spraggs-Hughes A, McAndrew CM, Ricci WM, Gardner MJ (2019). Bone marrow aspirate concentrate with cancellous allograft versus iliac crest bone graft in the treatment of long bone nonunions. OTA Int.

[5] Dimitriou R, Mataliotakis GI, Angoules AG, Kanakaris NK, Giannoudis PV (2011). Complications following autologous bone graft harvesting from the iliac crest and using the RIA: a systematic review. Injury.

[6] Moghaddam-Alvandi A, Zimmermann G, Büchler A, Elleser C, Biglari B, Grützner PA, Wölfl CG (2012). Results of nonunion treatment with bone morphogenetic protein 7 (BMP-7). Unfallchirurg.

[7] Dai J, Li L, Jiang C, Wang C, Chen H, Chai Y (2015). Bone morphogenetic protein for the healing of tibial fracture: a meta-analysis of randomised controlled trials. PLoSOne.

[8] Page MJ, McKenzie JE, Bossuyt PM, Boutron I, Hoffmann TC, Mulrow CD (2021). The PRISMA 2020 statement: an updated guideline for reporting systematic reviews. BMJ.

[9] Stang A (2010). Critical evaluation of the Newcastle-Ottawa scale for the assessment of the quality of nonrandomized studies in meta-analyses. Eur J Epidemiol.

[10] Takemoto R, Forman J, Taormina DP, Egol KA (2014). No advantage to rhBMP-2 in addition to autogenous graft for fracture nonunion. Orthopedics.

[11] von Ruden C, Morgenstern M, Hierholzer C, Hackl S, Gradinger FL, Woltmann A, Buhren V, Friederichs J (2016). The missing effect of human recombinant bone morphogenetic proteins BMP-2 and BMP-7 in surgical treatment of aseptic forearm nonunion. Injury.

[12] von Rüden C, Morgenstern M, Friederichs J, Augat P, Hackl S, Woltmann A, Bühren V, Hierholzer C (2016). Comparative study suggests that human bone morphogenetic proteins have no influence on the outcome of operative treatment of aseptic clavicle non-unions. Int Orthop.

[13] Hackl S, Hierholzer C, Friederichs J, Woltmann A, Buhren V, von Ruden C (2017). Long-term outcome following additional rhBMP-7 application in revision surgery of aseptic humeral, femoral, and tibial shaft nonunion. BMC MusculoskeletDisord.

[14] Liu D, Bai Y, Zhuang X, Lu S, Wang H. Comparison of effect of two different methods of postoperative bone nonunion. Xi Nan Guo Fang Yi Yao. 2019;29(10):1008–10 **(in Chinese)**

[15] Schmidmaier G, Moghaddam A (2015). Long Bone Nonunion. Z Orthop Unfall.

[16] Ban I, Troelsen A (2016). Risk profile of patients developing nonunion of the clavicle and outcome of treatment–analysis of fifty fivenonunions in seven hundred and twenty nine consecutive fractures. Int Orthop.

[17] Kwong FN, Harris MB (2008). Recent developments in the biology of fracture repair. J Am AcadOrthop Surg.

[18] Oryan A, Alidadi S, Moshiri A, Bigham-Sadegh A (2014). Bone morphogenetic proteins: a powerful osteoinductive compound with non-negligible side effects and limitations. BioFactors.

[19] Krishnakumar GS, Roffi A, Reale D, Kon E, Filardo G (2017). Clinical application of bone morphogenetic proteins for bone healing: a systematic review. Int Orthop.

[20] Tressler MA, Richards JE, Dmitri Sofianos F, Comrie K, Kregor PJ, Obremskey WT (2011). Bone morphogenetic protein-2 compared to autologous iliac crest bone graft in the treatment of long bone nonunion. Orthopedics.

[21] Friedlaender GE, Perry CR, Cole JD, Cook SD, Cierny G, Muschler GF, Zych GA, Calhoun JH, LaForte AJ, Yin S (2001). Osteogenic protein-1 (bone morphogenetic protein-7) in the treatment of tibial nonunions. J Bone Joint Surg Am.

[22] Cook SD (1999). Preclinical and clinical evaluation of osteogenic protein-1 (BMP-7) in bony sites. Orthopedics.

[23] Giannoudis PV, Einhorn TA, Marsh D (2007). Fracture healing: the diamond concept. Injury.

[24] Giorgio Calori M, Capanna R, Colombo M, De Biase P, O'Sullivan C, Cartareggia V, Conti C (2013). Cost effectiveness of tibial nonunion treatment: a comparison between rhBMP-7 and autologous bone graft in two Italian centres. Injury.

[25] Dahabreh Z, Calori G, Kanakaris N, Nikolaou V, Giannoudis P (2009). A cost analysis of treatment of tibial fracture nonunion by bone grafting or bone morphogenetic protein-7. Int Orthop.

[26] Haubruck P, Tanner MC, Vlachopoulos W, Hagelskamp S, Miska M, Ober J, Fischer C, Schmidmaier G (2018). Comparison of the clinical effectiveness of Bone Morphogenic Protein (BMP) -2 and -7 in the adjunct treatment of lower limb nonunions. Orthop Traumatol Surg Res.

[27] Lyon T, Scheele W, Bhandari M, Koval KJ, Sanchez EG, Christensen J, Valentin A, Huard F (2013). Efficacy and safety of recombinant human bone morphogenetic protein-2/calcium phosphate matrix for closed tibial diaphyseal fracture: a double-blind, randomized, controlled phase-II/III trial. J Bone Joint Surg Am.

[28] Govender S, Csimma C, Genant HK (2002). Recombinant human bone morphogenetic protein-2 for treatment of open tibial fractures: a prospective, controlled, randomized study of four hundred and fifty patients. J Bone Joint Surg Am.

